# Deciphering the Mechanism of Action Involved in Enhanced Suicide Gene Colon Cancer Cell Killer Effect Mediated by Gef and Apoptin

**DOI:** 10.3390/cancers11020264

**Published:** 2019-02-23

**Authors:** Blanca Cáceres, Alberto Ramirez, Esmeralda Carrillo, Gema Jimenez, Carmen Griñán-Lisón, Elena López-Ruiz, Yaiza Jiménez-Martínez, Juan A. Marchal, Houria Boulaiz

**Affiliations:** 1 Motril Health Center, Hospital Santa Ana, Motril, 18600 Granada, Spain; blcagr79@gmail.com; 2 Biopathology and Medicine Regenerative Institute (IBIMER), University of Granada, 18016 Granada, Spain; alrari80@gmail.com (A.R.); esmeral@ugr.es (E.C.); gemajg@ugr.es (G.J.); glcarmex@gmail.com (C.G.-L.); elruiz@ujaen.es (E.L.-R.); yaijmartinez@correo.ugr.es (Y.J.-M.); jmarchal@ugr.es (J.A.M.); 3 Biosanitary Institute of Granada (ibs. GRANADA), SAS-Universidad de Granada, 18016 Granada, Spain; 4 Department of Human Anatomy and Embryology, University of Granada, 18016 Granada, Spain; 5 Research Unit “Modeling Nature” (MNat), University of Granada, 18016 Granada, Spain; 6 Department of Health Sciences, University of Jaén, E-23071 Jaén, Spain

**Keywords:** *gef* gene, *apoptin* gene, combined therapy, apoptosis, caspase 3, caspase 8, caspase 9, necrosis, pore

## Abstract

Despite the great advances in cancer treatment, colorectal cancer has emerged as the second highest cause of death from cancer worldwide. For this type of tumor, the use of suicide gene therapy could represent a novel therapy. We recently demonstrated that co-expression of *gef* and *apoptin* dramatically inhibits proliferation of the DLD-1 colon cell line. In the present manuscript, we try to establish the mechanism underlying the enhanced induction of apoptosis by triggering both *gef* and *apoptin* expression in colon tumor cells. Scanning microscopy reveals that simultaneous expression of *gef* and *apoptin* induces the apparition of many “pores” in the cytoplasmic membrane not detected in control cell lines. The formation of pores induced by the *gef* gene and accentuated by apoptin results in cell death by necrosis. Moreover, we observed the presence of apoptotic cells. Performing protein expression analysis using western blot, we revealed an activation of mitochondrial apoptosis (increased expression of Pp53, cytochrome c, Bax, and caspase 9) and also the involvement of the extrinsic pathway through caspase 8activation. In conclusion, in this manuscript we demonstrate for the first time that the extrinsic pathway of apoptosis and pore formation is also involved in the cell death caused by the co-expression of the *gef* and *apoptin* genes. Our results suggest that co-expression of *gef* and *apoptin* genes induces an increase in post-apoptotic necrotic cell death and could be a valuable tool in the design of new antitumor strategies focused on the enhancement of the immune response against cancer cell death.

## 1. Introduction

Colorectal cancer is known to be the second most frequent cause of cancer death and the third in terms of incidence for both sexes combined. The estimation of new colorectal cancer cases in 2018 is over 1.8 million, and 881,000 patients are estimated to have died in 2018. These numbers represent about 1 out of 10 cancer deaths by this disease [1]. Surgical resection is the first-line treatment for localized early-stage colon cancer and adjuvant therapy is mainly used for high-risk colon cancer patients to increase the chance of cure. While multimodality therapies are a potential cure for low-metastatic liver and lung risk patients, palliative systemic therapy is aimed at improving the quality of life of non-surgical colon cancer candidates, prolonging the life expectancy of these patients. Drug resistance develops in almost all patients with colon cancer, which leads to a decrease in the therapeutic efficacy of anticancer agents and the urgent need for new alternative treatments [2,3]. The use of gene therapy aimed at delivering genetic material to cancer cells for therapeutic purposes seems to be a good alternative [4].

The use of toxic proteins encoded by killer genes delivered to cancer cells have been proposed as a promising tool for antitumor gene therapy. The main advantage of using these proteins is the ability to kill even quiescent tumor cells, while the classic genes used in conventional suicide gene therapy only target rapidly dividing cells by disrupting the DNA synthesis. Numerous suicide genes of different viruses, bacteria, and plants have been successfully used as a tool for this purpose in experiments aimed at killing cancer cells [5,6]. The anticancer effect of the toxin streptolysin O secreted by bacteria from the genus Streptococcus has been described both in vitro and in vivo [5,7]. Diphtheria toxin, ricin derived from plants, and pseudomonas exotoxin have an effective ADP-ribosylate elongation factor 2, and therefore, block the translation machinery of target cells and induce potent cell death. The potential use of this toxin to eradicate tumoral cells has been tested in different experiments [8,9,10,11]. The ability of *Apoptin*, a small protein encoded by chicken anemia virus to target selectively tumoral cells with no effect on normal cells (i.e., melanoma, hepatoma, different types of colon cancer, osteosarcoma, and cancers of breast, colon, prostate, lung, stomach, and cervix, among others) [12] could be used to induce apoptosis in cancer cells. Another candidate to be used is the *gef* gene. This gene expressed in *E. coli* encodes small and toxic proteins of approximately 50 amino acids that are able to induce apoptosis, cell cycle arrest, and the apparition of morphologic changes in a variety of human cancer cells [13,14,15]. We previously reported that the use of the combined antitumor effectof both *gef* and *apoptin* genes on human colon tumoral cells improved the anticancer effect of the single-suicide gene therapy. The synergistic anticancer effects of this double-suicide gene therapy overcome the deficient apoptosis induction found in advanced or metastatic colon cancer. In addition, the synergistic expression of both genes increased cell cytotoxicity by enhancing cell necrosis [16]. In the present study, we analyze the mechanism by which death occurs when *gef* and *apoptin* genes are expressed alone or combined.

## 2. Results

### 2.1. Morphological Findings

**Figure 1:**
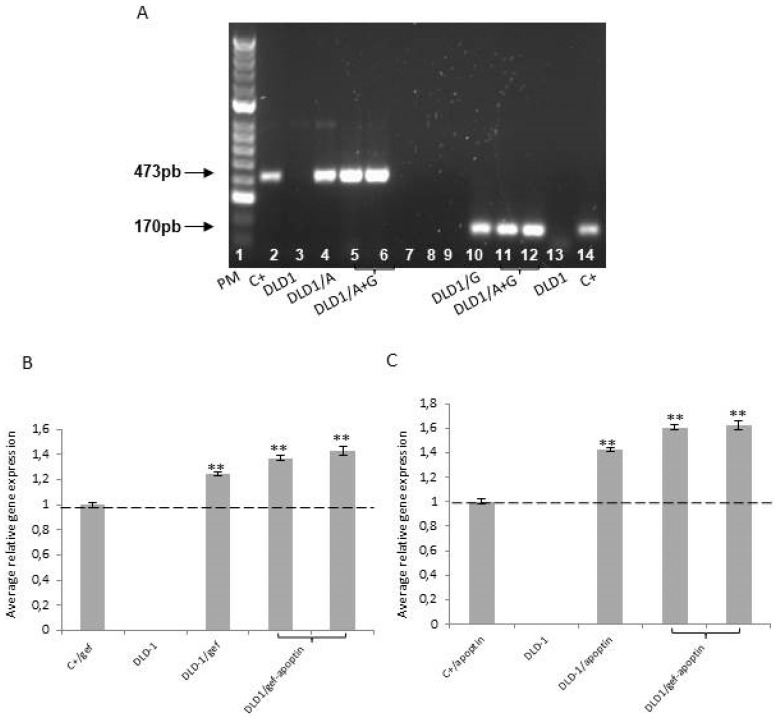
(**A**) RT-PCR analysis shows expression of *apoptin* and *gef* genes. Lane 1: hyper ladder II; lane 2: pRevTRE-apoptin (positive control); lane 3: DLD-1; lane 4: DLD1/apoptin; lane 5–6: DLD1/gef-apoptin; lane 10: DLD-1/gef; lane 11–12: DLD1/gef-apoptin; lane 13: DLD-1; lane 14: pRevTRE-gef (positive control). Average gene expression of *gef* (**B**) and *apoptin* (**C**) genes compared to the respective positive controls from left to right. pRevTRE-apoptin (positive control); DLD-1; DLD1/apoptin; DLD1/gef-apoptin; DLD-1/gef; DLD1/gef-apoptin; DLD-1; pRevTRE-gef (positive control). Data expressed as a mean ± SD from three independent experiments performed in duplicate (** *p* < 0.01 vs. control).

After 24 h treatment of control and transfected cells with Dox, RT-PCR was performed to detect *gef* and/or *apoptin* expression. Figure 1 shows the expression of *gef*, apoptin, or both genes, respectively, in the different cell lines (DLD1/Tet-On-*gef*, DLD1/Tet-On-*apoptin* and DLD1/Tet-On-*gef*-*apoptin*). No expression of *gef* or *apoptin* was detected in the control cell line (DLD-1).

**Figure 2:**
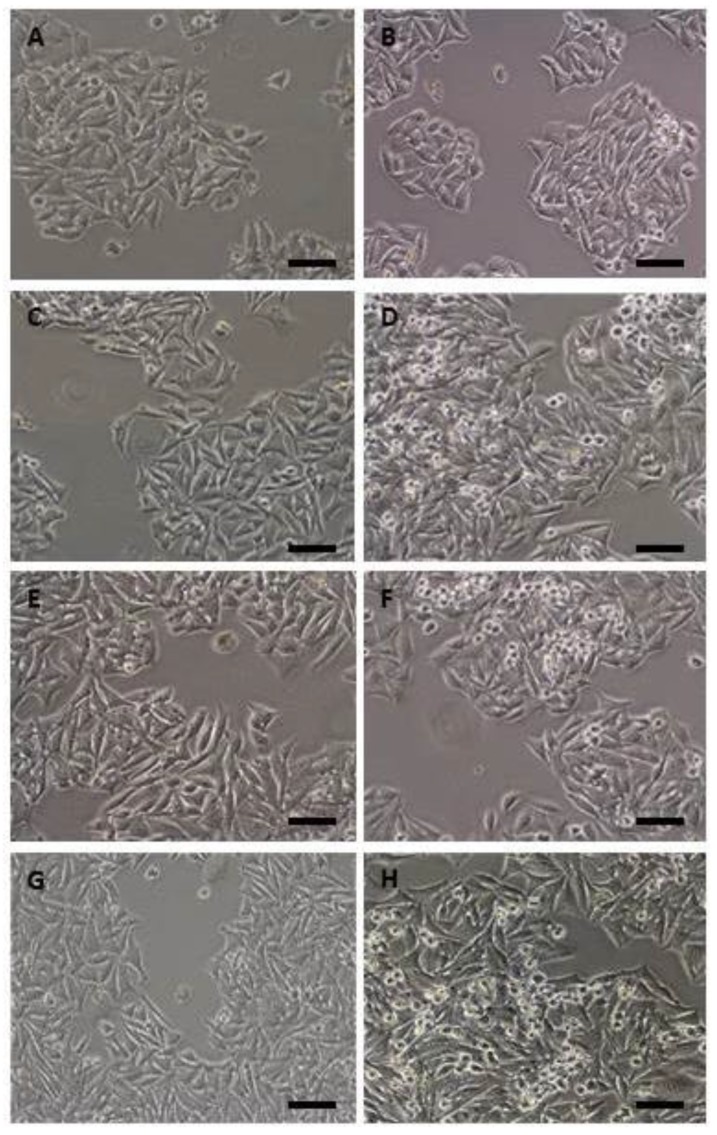
Phase contrast micrographs of parental DLD-1, DLD1/ Tet-On-*gef*, DLD1/ Tet-On-*apoptin*, and DLD1/ Tet-On-*gef*-*apoptin* cells after 48h of treatment with doxycycline (Scale bar = 100 μm). No differences were observed between DLD1cells (**A**) and cells treated with Dox (**B**). However, the lines transduced with *gef* and/or *apoptin* and induced with Dox were characterized by the presence of a group of smaller, rounded cells (more evident in D, F, H), less adhered to the culture bottle when compared to the parental cells. (**C**) DLD1/Tet-On-*apoptin* not induced with Dox; (**D**) DLD1/Tet-On-*apoptin* induced with Dox; (**E**) DLD1/Tet-On-*gef* not induced with Dox; (**F**) Tet-On-*gef* induced with Dox; (**G**) DLD1/Tet-On-*gef*-*apoptin* not induced with Dox; (**H**) DLD1/Tet-On-*gef*-*apoptin* induced with Dox.

The morphological changes induced by the genes in control (DLD-1) and transduced cells (DLD1/Tet-On-*gef*, DLD1/Tet-On-*apoptin* and DLD1/Tet-On-*gef*-*apoptin)* were analyzed using both optical and scanning microscopy. Light microscopy showed that all the cell lines were characterized by an epithelial morphology (Figure 2). We observed that the doses of Dox used to induce gene expression have no effect on DLD-1 cell morphology (Figure 2A,B) and colonies developed concentrically to the cells where their formation began. This finding was conditioned in part by the fact that dividing cells rarely separated from the monolayer. However, all the cell lines transduced and induced with doxycycline were characterized by the presence of cell shrinkage (Figure 2C–H). The size of these cells was smaller, showed a dense cytoplasm, and their organelles were more tightly packed. 

**Figure 3:**
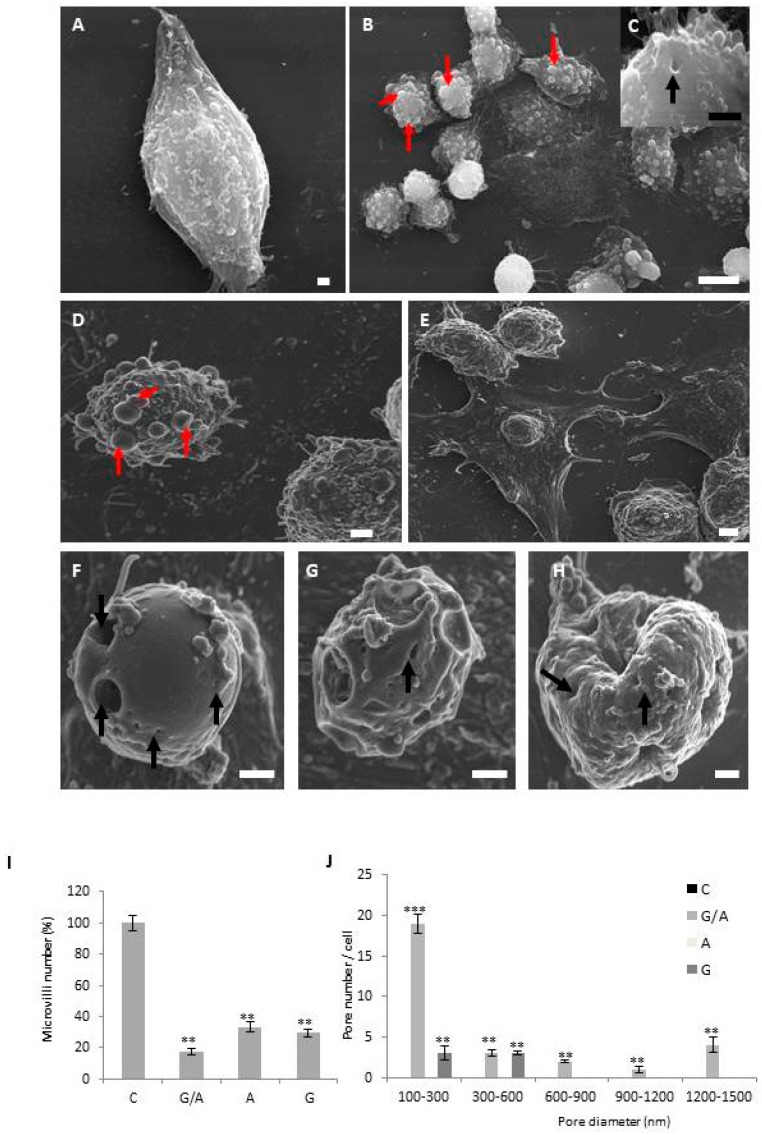
Scanning electron microscopic images of parental and transduced DLD-1 cells. DLD-1 cell line adhered to the culture surface with multiple filipodium (**A**). DLD1/Tet-On-gef cell line showed cells with several apoptotic bodies (red arrows) (**B**) and some cells have pores on their membrane surface (**C**). DLD1/Tet-On-apoptin cells showed signs of apoptosis with cytoplasmic membrane disruption, many apoptotic bodies, and clear signs of substrate detachment (**D**). DLD1/Tet-On-gef-apoptin cells, like the others, produced dead cells without filipodium and abundant apoptotic cells with the surface covered with apoptotic bodies (**E**). The most curious thing is that many more cells appeared with the surface full of pores of different sizes (**F**–**H**). Red arrows signal apoptotic bodies and black arrows pores (Scale bar A, C, D, F, G, H = 1 μm; Scale bar B = 10 μm; Scale bar E = 2 μm). Quantification of filipodium number (**I**). Pore number (**J**). The height of each bar represents the number of pores with diameters in the interval between its lower and upper bounds on the x-axis. Pore diameters between 100–1500 nm can be observed in DLD21/Tet-On-*gef-apoptin* cells. Quantifications were measured from the SEM images (5 random photos) from three independent sample preparations. The data is represented as mean ± S:D (** *p* < 0.01 vs. control and *** *p* < 0.001 vs. control).

Scanning electron microscopy analysis of parental DLD-1 cells showed cells adhered to the surface of the culture flask with the presence of elongated and/or more or less polygonal forms with their surface covered by multiple microvillous extensions (Figure 3A,B). The induction of the expression of *gef* and/or *apoptin* genes in the DLD-1 cell line caused severe morphological changes. In fact, most of the cells expressing both genes displayed initial signs of detachment of the substrate with cellular elements that remained attached to the culture surface only by thin cytoplasmic processes (Figure 3C,F,H). However, the most striking feature was the appearance in DLD1/Tet-On-*gef*-*apoptin* cells of many “pores” (Figure 3G,K–M) in the cytoplasmic membrane that were not detected in the DLD-1 parental cells or in those transduced with only *apoptin*. A smaller number of cells showed this feature in the line that expressed the *gef* gene (Figure 3E). Likewise, an important loss of filipodium in general was also observed in the three transfected cell lines (Figure 3D,E,G,K–M) in comparison to the control (Figure 3A,B). The presence of apoptotic bodies in all transduced and induced lines (Figure 3C–E,G) was a characteristic.

**Figure 4:**
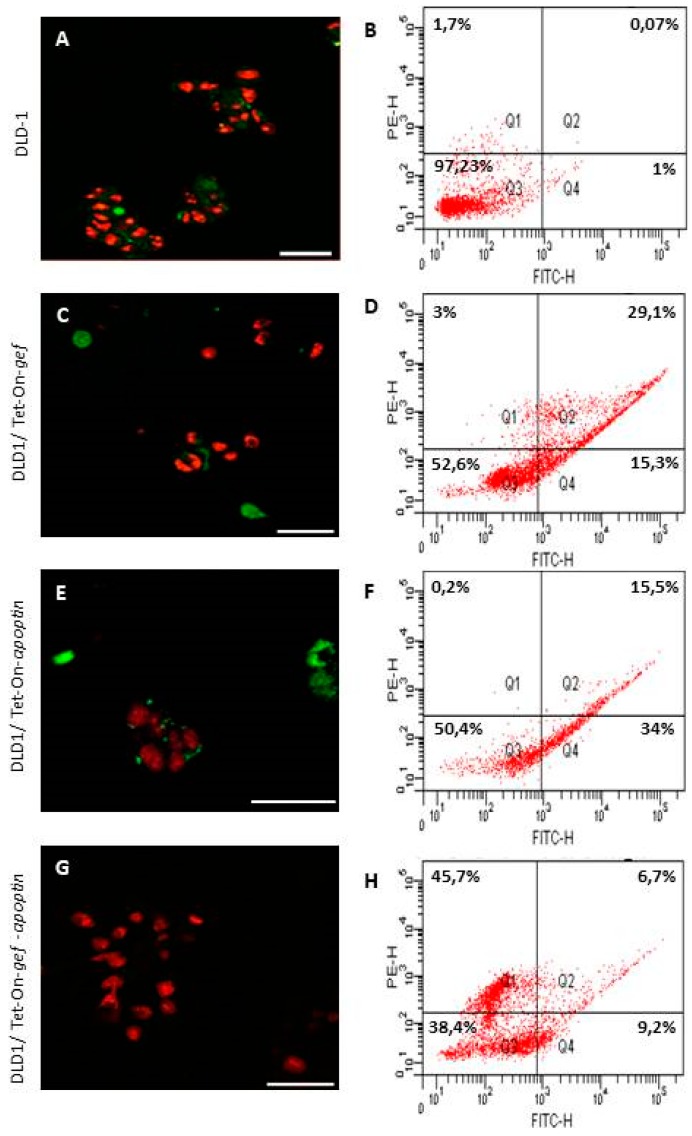
Confocal analysis and cell sorting quantification of DLD-1 (**A**,**B**), DLD1/Tet-On-*gef* (**C**,**D**), DLD1/Tet-On-*apoptin* (**E**,**F**), and DLD1/Tet-On-*gef*-*apoptin* (**G**,**H**) cells (Scale bar = 50 μm). Cells were stained with annexin V-FITC and PI. Early apoptosis (Q4: green stain for annexin-FITC), late apoptosis (Q2: green stain for annexin-FITC in combination with red stain for PI), and necrosis (Q1: red stain for PI) can be observed; viable cells are not visible (Q3). In DLD1 expressing *gef* or *apoptin,* cells in early and late apoptosis are observed (**C**,**E**). DLD1/Tet-On-*gef*-*apoptin* cells showed a higher proportion of necrotic cells when compared to some early and late apoptotic cells (**G**,**H**).

Confocal and flow cytometry analysis confirmed these observations. After Annexin V and IP staining, and in comparison with control cells (Figure 4A,B), DLD1expressing *gef* or *apoptin* genes showed cells in early and late apoptosis (Figure 4C–F). However, DLD1/Tet-On-*gef*-*apoptin* cells were stained mainly as necrotic cells (Figure 4G,H).

### 2.2. Mechanism of Action of Gef and/or Apoptin

To identify the mechanism by which *gef* and/or *apoptin* genes induce cell death, caspases 3, 9 and 8, cytochrome c, bax, and p53 were studied. Protein collection was performed after 2 days of induction with Dox, as described in material and methods. 

#### 2.2.1. Pp53

**Figure 5:**
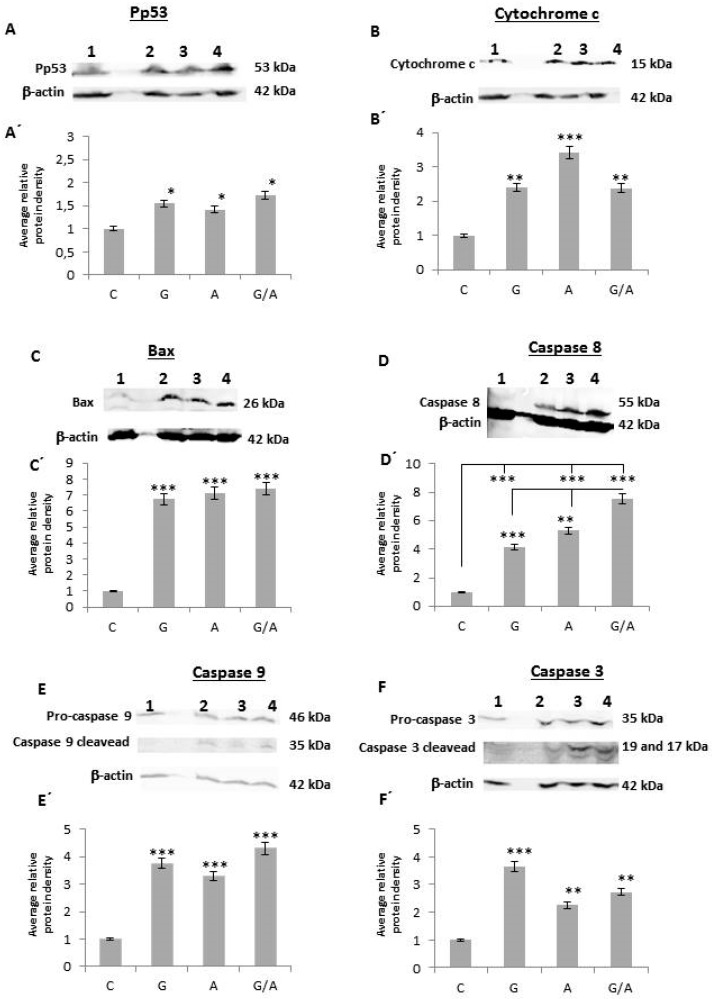
Western blot analysis of (**A**) Pp53, (**B**) caspase 8, (**C**) cytochrome c, (**D**) Bax, (**E**) caspase 3, (**F**) caspase 9. Line 1. Control DLD-1, line 2. DLD1/Tet-On-*gef*, line 3. DLD1/Tet-On-*apoptin*, line 4. DLD1/Tet-On-*gef*-*apoptin*. Relative quantification of the western blot proteins normalized with β-actin signal and relative to mock-treated cells (value 1) (**A**’, **B**’, **C**’, **D**’, **E**’, **F**’). Data expressed as a mean ±SD from three independent experiments performed in duplicate (* *p* < 0.05 vs. control, ** *p* < 0.01 vs. control and *** *p* < 0.001 vs. control).

We analyzed the expression of p53 under its phosphorylated form (Pp53). After normalization and comparison to the relative value of the control (1), we observed a clear increase in Pp53 expression in all the transduced lines but to different degrees (Figure 5A,A’). Thus, the band corresponding to DLD1/Tet-On-*gef* had a value of 1.55, DLD1/Tet-On-*apoptin* 1.42, and DLD1/Tet-On-*gef*-*apoptin* 1.72, which indicates that p53 was phosphorylated to a greater degree when *gef* and *apoptin* were expressed together.

#### 2.2.2. Cytochrome c

The release of cytochrome c to the cytosol was manifested in an increase in the intensity of the bands in the transduced and induced cell lines with respect to the corresponding control cell line (Figure 5B,B’). Taking the value 1 as the normalized value of the control cell line, the normalized values for the rest of the cell lines were: 2.4 for DLD1/Tet-On-*gef* cells, 3.41 for DLD1/Tet-On-*apoptin*, and 2.38 for DLD1/Tet-On-*gef*-*apoptin* cell line.

#### 2.2.3. Bax

The activation of Bax was manifested as an increase in the intensity of the bands in the transduced and induced cell lines in comparison to the band corresponding to the control cell line (Figure 5C,C’). The normalized and relativized values with respect to the control for the lines DLD1/Tet-On-*gef*-*apoptin*, DLD1/Tet-On-*apoptin*, and DLD1/Tet-On-*gef* were 7.4, 7.11, and 6.74, respectively.

#### 2.2.4. Caspase 8

The normalized values corresponding to the expression of caspase 8 in the lines DLD1/Tet-On-*gef*-*apoptin*, DLD1/Tet-On-*apoptin*, and DLD1/Tet-On-*gef* were 2.58, 1.81, and 1.66, respectively (Figure 5D,D’). As a result of the combined effect of both genes, caspase 8 was expressed to a greater degree in the cell lines infected by *gef* and *apoptin* together.

#### 2.2.5. Caspase 9

The normalized values corresponding to the expression of pro-caspase 9 in the lines DLD1/Tet-On-*gef*-*apoptin*, DLD1/Tet-On *apoptin*, and DLD1/Tet-On-*gef* were 1.32, 1.13, and 1.32, respectively (Figure 5E,E’). There is therefore a greater expression in the transduced and induced lines with Dox. The analysis of the activated form of caspase 9, using an antibody that detects its rupture, as described in material and methods, confirmed the results objectifying a greater increase of expression with respect to the control line. The normalized values were 4.31 for the line expressing both genes, 3.28 for the line with the *apoptin* gene, and 3.75 for the line with the *gef* gene.

#### 2.2.6. Caspase 3

The expression of pro-caspase 3 and its activated form was studied (Figure 5F,F’). The values obtained for the lines transduced and induced with Dox for pro-caspase 3 were 2.73 for DLD1/Tet-On-*gef*-*apoptin*, 2.25 for DLD1/Tet-On-*apoptin*, and 3.66 for DLD1/Tet-On-*gef*. For the activated form of caspase 3 the values were 2, 2.89, and 2.15, respectively. The expression of the genes has therefore caused activation, in general terms, at least twice as much with caspase 3 as compared to the control line.

After the analysis of the data, it is evident that there is an activation of mitochondrial apoptosis (increased expression of Pp53, cytochrome c, Bax, and caspase 9), but we also observed an activation of caspase 8 that would correspond to the extrinsic pathway of the apoptosis.

## 3. Discussion

The role of toxins in cancer treatment has been revealed due to their ability to kill tumoral cells efficiently, making them a promising tool to be used as potential anticancer molecules. Among others, toxins encoded by bacteria (i.e., Corynebacterium or Pseudomonas species) or a plant belonging to the ricin family have been successfully used to achieve cancer cell eradication [8,9,10,11]. Apoptin has been shown to induce apoptosis in a selective manner in tumoral cells while not exerting a toxic effect in normal cells. The *gef* gene from Escherichia coli shows an antiproliferative effect in different cancer cell types (i.e., breast, melanoma, and colon cancer cells) [13,14,17]. Moreover, the use of combined gene therapy improves the results obtained using single-gene therapies, enhancing the efficacy and overcoming the shortcomings of this type of therapy. Thanks to these improved results, combination gene therapy has become a first-line research field in gene therapy [7,18,19]. We recently demonstrated that the combined transduction of *gef* and *apoptin* is able to target colon cancer cells, highlighting the improved anticancer effect by using two direct suicide genes as a double-suicide gene therapy. Simultaneous expression of *gef* and *apoptin* achieve a synergistic effect by decreasing tumoral cell viability, enhancing necrosis, and increasing apoptosis induction. In our previous publication, results showed an improved effect of the double-suicide *gef* and *apoptin* genes to decrease membrane potential measured in mitochondria using a JC-1 probe after 2 and 6 days of doxycycline treatment compared to single *gef-* or *apoptin* treated cells and control cells, suggesting apoptosis induction in colon tumoral cells occurs via mitochondrial pathways [16]. In the present study, we have carried out morphological studies and analyzed the protein profiles of several genes involved in different pathways of apoptosis to try to establish the mechanism of action of *gef* and *apoptin* genes alone or combined.

Analysis using optical microscopy of the DLD-1 colon carcinoma cell line transduced with the *gef* and/or *apoptin* genes and induced with Dox showed, in addition to small cells with an epithelial morphology, which were similar to the parental cells, enshrined flat cells. These cells were smaller in size, their cytoplasm was dense, and their organelles were more tightly packed. This cell group was previously described as apoptotic cells by [20] in the B16F10 cell line of murine melanoma treated with ganciclovir after its transfection by the simple herpes virus. Apoptosis has been extensively studied and classified following morphological criteria [21]. This phenomenon is characterized by membrane blistering, the adoption of a round shape by cells, condensation of cytoplasm and chromatin, and the formation of apoptotic bodies, among other well-known phenomena [22]. In order to study the effect of *gef* and *apoptin* expression on treated cells at morphological level, we carried out SEM microscopy to obtain high-resolution micrographs of treated cells. SEM microscopy allowed us to perform a thorough study of the cell surface. Our results provided interesting data regarding the cell death induced by *gef* and/or *apoptin*. On the one hand, we have been able to observe an important loss of filipodium in general in the three transfected cell lines in comparison to the DLD-1 control line. In addition, other signs of cellular suicide induction were detected, including the formation of apoptotic bodies, membrane blebbing, or cell contraction. Apoptotic bodies were found in *gef* and *apoptin* transduced and induced cell lines but to a greater extent in the line that co-expresses both genes. Apoptosis induction is related to cytoplasmatic and DNA contraction and also with the apparition of membrane blebbing [19]. Also well-known is the involvement of ROCK1 protein in cell membrane disorganization by caspase-mediated activation and its role in actin stabilization, myosin phosphorylation, and the final coupling of actin-myosin cytoskeleton to the plasma membrane. Through this mechanism of action, ROCK1 eventually leads to membrane blebbing and cell contraction [23,24]. Similar morphological changes were observed in breast cancer due to *gef* gene expression [25] and in lung and colon cancer cells after 5-fluorouracil-loaded calcium phosphate nanoparticle treatment [26]. This data is in agreement with our previous publication in which we analyzed, using flow cytometry with annexin V and propidium iodide and transmission electron microscopy, the effect of *gef* and *apoptin* genes on the DLD-1 cell line.

However, the most striking feature was the appearance of many “pores” in the cytoplasmic membrane, which were not detected, at any time, in the DLD-1 parental cells or in those transduced only with *apoptin*, and in smaller measure in the line that expresses *gef* gene alone. It has been described that in prokaryotic cells the expression of the *gef* gene leads to an efflux of intracellular Mg^2+^ and an influx of periplasmic molecules by decreasing membrane potential [27]. This phenomenon finally induces cell death due to morphological changes [28]. Not with standing that *gef* gene mechanisms of action have not been elucidated, SEM microscopy studies carried out in MS-36TG melanoma cells expressing this gene showed the apparition of pore-like structures in the cell membrane, maybe related to *gef* expression in this cancer cell line [15]. Interestingly, the number of pores observed through the *gef* gene induction was much less than that observed with the co-expression of the *gef* and *apoptin* genes. This suggests that although *apoptin* is not a pore-forming protein, it multiplies the pore-forming capacity of the *gef* gene in eukaryotic cells, which would explain the synergistic antiproliferative effect of both genes. In fact, we have previously demonstrated that simultaneous expression of both *gef* and *apoptin* genes enhances cell cytotoxicity by cell necrosis induction [16]. Necrosis characterized by a disorganization of the plasma membrane that finally leads to changes in osmolarity provoking cell swelling and the release of cytosolic constituents into the extracellular space, initiating an inflammatory response in some cases. Nevertheless, in necrosis the nucleus appears to be well preserved at the beginning of the stage compared to apoptosis [29,30,31,32]. The excessive pore formation induced by *gef* gene, and accentuated by *apoptin,* results in cell death by necrosis. Although cell membrane structure is promptly disorganized in necrosis, in apoptosis it is initially kept intact in the early stages [30,33]. This manner of death has also been described as a mechanism of action of several bacterial pore-forming toxins such as *Bacillus thuringiensis* and *Clostridium perfringens* delta-toxin [34,35,36].

The effects of the *gef* and *apoptin* genes alone or combined to induce cell death were also assessed using confocal microscopy with FITC-conjugated annexin V and PI stains. In the DLD-1 cell line expressing *gef* or apoptin, we detected cells in early and late apoptosis. The proportion of DLD1/Tet-On-*gef*-*apoptin* cells under necrosis death was higher than cells under early or late apoptosis. In the DLD-1 control cells, most of them were negative for both dyes, while a low number showed apoptosis features. This is particularly important in colon cancer development and the formation of metastases associated with apoptosis resistance [37]. Therefore, it is essential there is the presence of an alternative mechanism of cell death to ensure the cytotoxic effect of *gef* and *apoptin* genes to target cancer cells. Moreover, the increase in apoptosis and necrosis in uncontrolled proliferating cells was described in several antitumor treatments such as combined treatment with the monoclonal antibody IMC-C225 and the topoisomerase I irinotecan inhibitor (CPT-11) in DLD-1 cell line [38] and inhibition of long non-coding RNA PVT1 in human acute erythroleukemia cells [39]. 

Apoptosis can occur mainly through two different pathways: the extrinsic (by transmembrane receptors) and the intrinsic (mitochondrial). The extrinsic pathway requires trans-membrane receptors called “Death Receptors” (DR) and ligands that interact with such receptors. The receptors belong to the superfamily of tumor necrosis factor (TNF) receptors. It includes Fas (CD95), TNFR-I and II, p75NTR, DR-3, and TRAIL (DR-4 and DR-5). They have an intracellular domain (DD) and an extracellular domain rich in cysteines. Adapter proteins such as RIP, FADD, RAIDD, or TRADD bind to the intracellular domains to form the signaling complex that induces cell death. Once the complex is formed, initiating caspases 8 and 10are activated, and finally, the activation caspases 3, 6, and 7 are activated, which generates the signal to the endonucleases that will culminate in cellular apoptosis [40]. Bcl-2 family proteins play a key role in the mitochondrial cell death pathway by disrupting the mitochondrial membrane potential and the release of apoptogenic factors, such as cytochrome c from mitochondria, in the cytosol [41]. The Bcl-2 family consists of members that can be divided according to their activity into two groups: anti-apoptotic proteins such as Bcl-2, Bcl-x, BAG, Bcl-w, and Bcl-XL and pro-apoptotic proteins such as Bax, Bcl-xL, Bcl-xS, Bax, Bik, Bak, Blk, Bad,Bcl-10,and BimandBid [42].

Cell death induction exerted by *gef* or *apoptin* expression has been related to apoptosis intrinsic pathway [16,43]. We have previously demonstrated that simultaneous expression of both genes or each one alone induces the alteration of the mitochondrial membrane structure, suggesting the importance of the mitochondrial pathway in apoptosis mediation. In addition to the alterations of the membrane and DNA and protein degradation, the fundamental characteristic of apoptosis is mainly the activation of caspases, a family of proteins composed of cysteine proteases [44]. The activation of these proteins plays a key role in cytoskeleton and nuclear scaffold degradation by activation of other proteins. Additional to this process, they stimulate DNAse and enhance the degradation of the DNA [45]. In the present study, we carried out a western blot analysis of the caspases 3, 9, and 8, cytochrome c, bax, and p53 to dilucidate the mechanism of action of both genes in DLD-1 colon cancer cells. Data showed an increase inPp53 expression of cytochrome c, Bax, and caspases 9 after 48h of the induction of gene expression. This data supports the hypothesis that *gef* and *apoptin* are still capable of activating the mechanism of cell destruction mediated by the mitochondrial lesion. Thus, the activation of pro-apoptotic proteins induces pore formation in the mitochondrial outer membrane and the release of proteins such as the serine protease HtrA2/Omi cytochrome-c, Smac/DIABLO, or cytochrome-c by the diminution of the mitochondrial transmembrane potential. In the apoptosome formation, the cytochrome c joins to procaspase-9 and the adapter molecule Apaf-1 to later activate procaspase-3 by hydrolysis and trigger the last stage of apoptosis [46]. That the activation of caspase-mediated apoptotic pathways is a strategy to induce the destruction of tumor cells is confirmed by the experiments of Jang and collaborators which demonstrate that transfection of ANT1 triggers apoptosis induction in the MDA-MB-231breast cancer cell line by inactivating NF-κB and increasing the expression of Bax [47]. The induction of ANT1 apoptosis was accompanied by the alteration of the mitochondrial membrane potential, the release of cytochrome c, and the activation of caspases 9 and 3. Other suicide genes, such as *cry1Ab* [48] and *gef* [49], suggest pore formation and activation of Protein Kinase A (PKA).

However, we have also observed a clear activation of caspase 8 when the *gef* and *apoptin* genes are expressed alone or in combination. This data suggests that apoptosis mediated by the expression of *gef* and *apoptin* in the DLD-1 line also involves molecules of the extrinsic pathway of apoptosis [42].

The execution phase is the endpoint where both the intrinsic and extrinsic pathways converge and is considered the final stage of apoptosis. The last stage of apoptosis is characterized by the activation of caspases which activates proteases and endonucleases that leads to cytoskeletal protein and nuclear material degradation. In this context, caspase-3 plays a key role as effector of the pathway by cleaving different substrates including plasma membranes, cytoskeletal and nuclear proteins, among others (alpha foldrin and NuMA proteins) [50]. Our results support the hypothesis that the convergence of both intrinsic and extrinsic pathways can induce the activation of caspase-3. Caspase-3 plays a key role in the executioner caspase pathway and is activated by any of the initiator caspases (caspase-8, caspase-9, or caspase-10).

**Figure 6:**
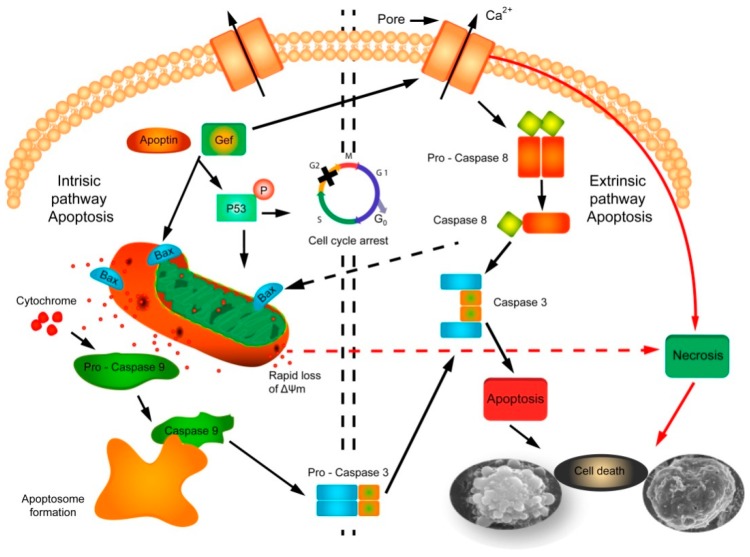
Schematic representation of cell death pathways induced by *gef/apoptin. gef/apoptin* expression induces a decrease in DLD-1 cell growth and viability by triggering necrosis and apoptosis through both the intrinsic and extrinsic apoptotic pathways. Cellular stress induces apoptosis intrinsic pathway and activates the Bcl-2 family of proteins. Pore formation is then promoted by BAX oligomers in the outer mitochondrial membrane and releases various proteins including cytochrome c. Procaspase-9 is recruited by the apoptosome through cytochrome c induction, inducing the dimerization and activation of caspase-9. Caspase-9 activation triggers caspase cascade and finally induces executioner caspases-3. These caspases are responsible for cleavage of numerous cellular proteins, leading to apoptosis. In the extrinsic caspase-8 dependent pathway there are two different ways. The type I pathway, where caspase-8 directly cleaves and activates caspase-3 leading to cell death, and the type II pathway, wherecaspase-8 activates BAX leading to mitochondrial pore formation and the activation of the caspase cascade culminating in caspase-3 activation.

Several authors have shown that the overlap and integration of the two pathways occurs at the Bid level, a proapoptotic gene belonging to the Bcl-2 family. The proapoptotic activity of Bid is greatly increased by its caspase-8-mediatedrupture and translocation to the mitochondria, where it promotes the activation of the caspase-3, the formation of the apoptosome, and the release of the cytochrome c [18,51]. ICAD caspase-3-mediated cleavage promotes the rupture of the complex CAD and it is inhibitor ICAD (CAD-ICAD complex) in apoptotic cells, while in proliferating cells the integrity of the structure is maintained [52]. Finally, CAD provokes chromosomal DNA degradation and chromatin condensation leading to the formation of apoptotic bodies by cytoskeletal disintegration [41,53,54,55]. The potential mechanism of action of gef/apoptin genes in DLD-1 tumor cancer cell line is summarized in Figure 6. 

Excessive pore formation induced by the *gef* gene in both mitochondrial and cytoplasmic membrane leads to target cell death by necrosis due to alteration of mitochondrial activity as a result of a drastic reduction of ATP and potassium levels. At lower concentrations (separate *gef* or *apoptin* expression), death may occur in a programmed way via apoptosis in its two pathways. However, co-expression of both genes subjects the target cell to an excessive load of toxins that implies the appearance of uncontrolled cell death or necrosis. This necrosis could correspond to the secondary post-apoptotic necrosis that, according to Silva (2010), is the natural result of the complete apoptotic program [56]. Recent studies have described that when there is a large number of apoptotic cells in vitro or when the phagocytic system of the organism is overwhelmed, the apoptotic cells progress to a stage of secondary necrosis in which they progressively lose their structural integrity and release parts of their cytoplasm due to the permeabilization of its plasma membrane [57,58]. In this context, the same phenomenon was observed in MCF-7 breast cancer cells after exposure to the *gef* gene combined with new synthesized cyclic and acyclic O,N-acetals compounds [59]. Moreover, Chen et al. observed that the infectious pancreatic necrosis virus induces post-apoptotic necrotic cell death through loss of mitochondrial membrane potential (MMP) followed by caspase-3 activation in CHSE-214 cells [60]. Furthermore, there are several studies that suggest the existence of common regulators between necrosis and apoptosis [58]. Hitomi and collaborators showed that TNFα activates signaling mediated by RIP 1 kinase for the induction of subsequent genes that influence either necrosis or apoptosis [61]. In our system, we believe that the combination of genes *gef* and *apoptin* induces a clear increase in post-apoptotic necrotic cell death which could facilitates intratumoral immune responses, as has been shown previously in breast cancer [62], and could be a valuable tool in the design of new antitumor strategies focused on the enhancement of the immune response against cancer cell death [63].

## 4. Material and Methods

### 4.1. Cell Lines

DLD-1 human colorectal carcinoma cells (ATCC^®^ CCL-221™, Manassas, VA, USA) were obtained from the Immunology Service, Virgen de las Nieves Hospital (Granada, Spain). These types of cells were originally isolated from an adult male with a Dukes’ type C colorectal adenocarcinoma where the cancer had spread to at least one lymph node in the area close to the bowel [64].

The DLD1/Tet-On-*gef*, DLD1/Tet-On-*apoptin*, and DLD1/Tet-On-*gef*-*apoptin* cell lines, and *gef* and/or *apoptin*-expressing colorectal carcinoma cell lines controlled using a Tet-on system and induced using doxycycline (Dox) were derivates from DLD-1 cells following methodology previously described by us [16].

DLD-1 cells were grown at 37 °C, 5% CO_2_ in Dulbecco’s modified Eagle medium (DMEM) (Sigma, St. Louis, MO, USA) in the presence of 10% heat-inactivated fetal bovine serum (FBS) (Sigma), 1% HEPES buffer, 40 mg/L gentamicin, 2% L-glutamine, 2.7% sodium bicarbonate and 500 mg/L ampicillin. Transfected DLD1 cell line with *gef*, *apoptin*, or both genes were cultured in the same conditions and medium described above supplemented with hygromycin B (0.4 mg/mL, Sigma) and doxycycline 0.2 mg/mL.

### 4.2. RNA Extraction Protocol and RT-PCR Analysis

Extraction of total RNA from DLD-1, DLD1/Tet-On-*gef*, DLD1/Tet-On-*apoptin*, and DLD1/Tet-On-*gef*-*apoptin* cells was carried out using the RNA extraction kit (Promega Corporation, Madison, WI, USA) after 24 h of induction with doxycycline (0.2 mg/mL). For the reverse-transcription of the RNA into cDNA, one microgram of RNA from each cell line was mixed with the cDNA synthesis kit (Promega). Reverse transcription (RT) was done as follows: each tube contained 0.4 µM of random hexamers, 1 µM of each deoxynucleotide triphosphate (dNTP), 2.0 mM MgCl2, 24 units of avian myeloblastosis virus reverse transcriptase (Promega), and 40 U RNasin (Promega) in a total volume of 20 µLof RT buffer. After incubation at 42 °C for 20 min and 94 °C for 5 min (Linus Autocycler 32, CulteK S.L., Madrid, Spain), samples were resuspended in 80 µL H2O with diethylpyrocarbonate.

PCRs were performed using 5 µLof the RT products with the following primers: *Gef* 1: 50 GAAGCAGCATAAGGCGATG 30 and *Gef* 2: 50 CCGCCGTTGCTCTTACTC 30; Apop 1: 50 GAACGCTCTCCAAGAAGATAC 30; and Apop 2: 50 ATTACCACTACTCCCAGCCG 30. PCR (*gef* 94 °C for 5 min, 51 °C for 1 min, and 72 °C for 1 min; *apoptin*: 48.9 °C for 5 min, 51 °C for 1 min, and 72 °C for 1 min) was carried out for 32 cycles, with a final elongation step of 72 °C for 10 min. For the product visualization, 10 µL of the reaction was loaded on a 2% agarose gel, detected using ethidium bromide staining, and the image was photographed under ultraviolet light. The integrity of the RNA was assessed by amplification of β-actin. The analysis of the intensity of the signal was carried out using the Quantity One program. The values of each band were relativized with respect to the control sample, to which the value 1 was assigned.

### 4.3. Scanning Electron Microscopy

The cells were grown on sterile 10 × 10 cm coverslips and then were fixed with 2.5% glutaraldehyde in 0.05 M cacodylate buffer, pH 7.4, containing CaCl_2_ 1.24 mM, KCl 0.7 M and MgCl_2_ 1.24 mM for 2 h at 40 °C with. After fixation, cells were washed with cold phosphate buffered salineand treated with 1% tannic acid in 0.1 M cacodylate buffer for 1 h at 4 °C. Then, the preparation was washed again briefly in the same buffer. After centrifugation in 1% agarose, cells were post-fixed using OsO_4_. Using amyl acetate/ethanolthe fixated cells were dehydrated and finally dried and coated with gold. Images were acquired using a Hitachi S-800 scanning electron microscope (SEM) (Hitachi, Tokyo, Japan). Counting of filipodium number and number of pores per cell were performed using ImageJ with quantification for each cell type from 5 random photos from three independent sample preparations.

### 4.4. Confocal Microscopy and Flow Cytometry for Apoptosis Detection

Cells were seeded at a confluence of 5 × 10^3^ cells/well in an8-well Labtek chamber slide. After 24 h, they were treated with Dox for 48 h. After that, culture medium was discarded and cells washed with cold phosphate buffered saline and incubated with both annexin V-FITC and propidium iodide solution for 15 min at room temperature. Finally, cells were washed with binding buffer and were processed using flow cytometry for its quantification. In addition, a sample was taken and preserved using mounting medium and cover slips before confocal microscopic imaging. Images were acquired using a Leica SP2 Confocal Microscope. Counting of filipodium number and number of pores per cell were performed using ImageJ with quantification for each cell type from 5 random photos from three independent sample preparations.

### 4.5. Protein Analysis

To study protein expression differences after 48 h of Dox treatment we performed a western blot. Cells seeded on 6-well plates were lysed using Laemmli buffer and the lisate was loaded in the electrophoresis gel, electroblotted onto nitrocellulose membranes and submerged in phosphate buffered saline containing 5%non-fat dried milk in agitation for 1h at room temperature. To detect protein expression, the following primary antibodies were used: phosphorylated p53 (p-p53 (FP3.2): sc- 51690, Santa Cruz Biotechnology, (Santa Cruz Biotechnology, Santa Cruz, CA, USA), caspase 8 (caspase-8 (8CSP03): sc-56070, Santa Cruz Biotechnology), caspase 3, 1:1000 in 5% non-fat dry milk in TBS (Cell Signaling, Beverly, MA, USA), caspase 9 (caspase-9 p35 (A-): sc- 133109, Santa Cruz Biotechnology), cytochrome-c (cytochrome c (7H8): sc- 13560, Santa Cruz Biotechnology), Bax, (Bax (N-20): sc- 493, Santa Cruz Biotechnology) and β-actin (A2228, Sigma). Secondary antibodies used included anti-rabbit IgG peroxidase conjugate (Sigma, A0545) and anti-mouse IgG peroxidase conjugate (Sigma, A9044). Protein–antibody complexes were made visible using enhanced chemiluminescence (ECL, Bonus, Amersham, Little Chalfont, UK) with the program IMAGE READER LAS-4000 in a LAS-4000 imaging system.

The analysis of the intensity of the signal was carried out using the Quantity One program. The values of each band were normalized by dividing them by the value of their β-actin, and they were relativized with respect to the control sample to which the value 1 was assigned.

### 4.6. Statistical Analysis

The data is expressed as means ± standard deviation (SD). The statistical significance of differences between the two groups was determined using Student’s *t* test. A *p* value of 0.05 or less was considered to be statistically significant.

## 5. Conclusions

The data obtained in this study demonstrates for the first time that that co-expression of *gef* and *apoptin* genes induces apoptosis, which occurs not only via intrinsic but also extrinsic signaling pathways. Both *gef* and *apoptin* genes induce an important increase of caspase 8 and 9 that converges at caspase 3 that acts as an “executor” of apoptosis. In fact, caspases 8 and 9 reached higher levels after the co-expression of the *gef* and *apoptin* genes than when expressed separately. This data could explain, at molecular level, the synergistic effect observed through the combination of *gef* and *apoptin* genes. These results are of interest because apoptosis in advanced or metastatic colon cancer may be deficient. Moreover, co-expression of *gef* and *apoptin* genes induces an increase in post-apoptotic necrotic cell death that could be a valuable tool in the design of new antitumor strategies focused on the enhancement of the immune response against cancer cell death.
